# Specialist nurse for organ donation in an emergency department will increase organ donation

**DOI:** 10.1186/cc12450

**Published:** 2013-03-19

**Authors:** T Neill, J Millar, P O'Connor, P Glover

**Affiliations:** 1 Royal Victoria Hospital, Belfast, UK

## Introduction

In the UK, three people die each days awaiting transplantation, due to the unavailability of donor organs. Traditionally, donor identification has been restricted to the ICU. However, following the UK Organ Donation Taskforce report in 2008 [1], a number of emergency departments (EDs) have been working with specialist nurses for organ donation (SN:OD) to identify potential donors and approach their families for consent in the ED. We present our initial experience after the introduction of a SN:OD to an Irish teaching hospital's ED.

## Methods

We conducted a retrospective review of deaths in our ED during a 28-month period. For those who died in the ED, case notes were reviewed to identify those suitable for organ donation. Referral and donation rates were compared in two cohorts, pre and post introduction of a SN:OD. Fisher's exact test was used to assess differences between groups.

## Results

Ninety-one deaths occurred in the study period. Following introduction of the SN:OD, referrals increased from zero to eight. Of the eight referred, three received consent and were transferred to the ICU, two of whom became successful donors. The number of missed potential donors fell from six to one (*P *= 0.009).

## Conclusion

Introduction of a SN:OD and a clinical pathway has led to the identification of previously missed potential organ donors in the ED. Several patients have subsequently been admitted to critical care solely to facilitate organ donation.
